# SUPERFACT: A Model Fuel for Studying the Evolution of the Microstructure of Spent Nuclear Fuel during Storage/Disposal

**DOI:** 10.3390/ma14216538

**Published:** 2021-10-30

**Authors:** Thierry Wiss, Oliver Dieste, Emanuele De Bona, Alessandro Benedetti, Vincenzo Rondinella, Rudy Konings

**Affiliations:** 1 Joint Research Centre, European Commission, P.O. Box 2340, 76125 Karlsruhe, Germany; oliver.dieste@kit.edu (O.D.); Emanuele.DE-BONA@ext.ec.europa.eu (E.D.B.); alessandro.benedetti@ec.europa.eu (A.B.); vincenzo.rondinella@ec.europa.eu (V.R.); rudy.konings@ec.europa.eu (R.K.); 2 Institute for Nuclear Waste Disposal (INE), Karlsruher Institute of Technology (KIT), Campus North, Hermann-von-HelmholtzPlatz 1, 76344 Eggenstein-Leopoldshafen, Germany

**Keywords:** alpha-damage, SUPERFACT irradiation, spent fuel, microstructure, helium

## Abstract

The transmutation of minor actinides (in particular, Np and Am), which are among the main contributors to spent fuel α-radiotoxicity, was studied in the SUPERFACT irradiation. Several types of transmutation UO_2_-based fuels were produced, differing by their minor actinide content (^241^Am, ^237^Np, Pu), and irradiated in the Phénix fast reactor. Due to the high content in rather short-lived alpha-decaying actinides, both the archive, but also the irradiated fuels, cumulated an alpha dose during a laboratory time scale, which is comparable to that of standard LWR fuels during centuries/millenaries of storage. Transmission Electron Microscopy was performed to assess the evolution of the microstructure of the SUPERFACT archive and irradiated fuel. This was compared to conventional irradiated spent fuel (i.e., after years of storage) and to other ^238^Pu-doped UO_2_ for which the equivalent storage time would span over centuries. It could be shown that the microstructure of these fluorites does not degrade significantly from low to very high alpha-damage doses, and that helium bubbles precipitate.

## 1. Introduction

Aside from plutonium, the radiotoxicity of spent fuel during the first centuries of storage/disposal is largely due to Minor Actinides (MA), particularly americium. [1].

In the case of spent fuel reprocessing and minor actinide separation, americium can be transmuted in fast reactors in so-called Minor Actinide Bearing Blankets (MABB), therefore reducing the global inventory of High Level Waste (HLW). An initial transmutation experiment in this sense was performed in the SUPERFACT irradiation project during the mid-1980s [2], when UO_2_-based fuels with a different content of americium, as well as other actinides, were irradiated in the Phénix fast reactor. The project investigated the possibility of transmuting those minor actinides into less dangerous elements and at the same time obtain energy from waste. Two different sets of samples were prepared: one set with a low content of MAs (homogeneous mode), and another with a high content of MA (heterogeneous mode). These samples were irradiated up to a burnup of 6.5 at% in the case of the homogeneous, and 4.5 at% for the heterogeneous.

Some SUPERFACT fuels recently examined by Transmission Electron Microscopy (TEM) showed remarkable behaviour in terms of radiation damage [3]. It could be shown that no micro-craking occurred despite a high level of damage and substantial radiogenic helium formation, in contrast with aged PuO_2_, for example. 

Samples from the SUPERFACT fuel with 20 at% americium and 20% neptunium content have been archived for 27 years since their production; irradiated rods have been sampled and post-irradiation examinations performed soon after the irradiation. Recently, both irradiated and archive samples have been analysed to study their different microstructures. All samples selected in this study have various compositions in terms of MA content but share the same fluorite structure. They also differ from real spent fuels in terms of the presence of fission products and the stoichiometry of some. Typically, with high MA content, materials are sub-stoichiometric. For the irradiated SUPERFACT fuel with a high MA content (designated SF14), it is assumed that the high temperature reached during irradiation anneals most defects, and the calculated damage (dpa) is that from end of irradiation (EOI) until the time of investigation. The damage level attained by this sample in particular is beyond anything previously studied for ^238^Pu-doped UO_2_ samples.

Electron Energy Loss Spectroscopy (EELS) was employed to determine the evolution of the composition of these materials to calculate the cumulated alpha dose. TEM/Scanning TEM (STEM), on the other hand, allowed one to examine the microstructure as a function of cumulated alpha dose.

**Figure 1:**
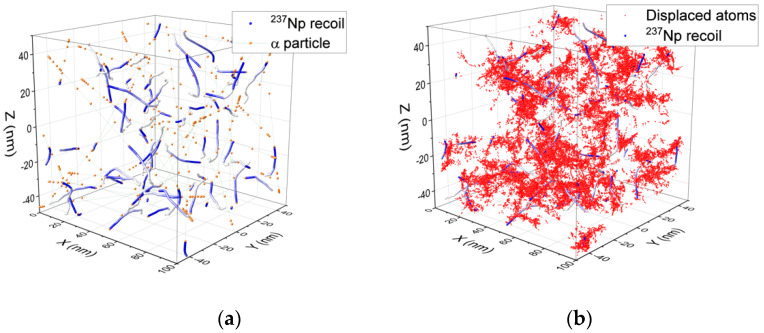
Simulation made with SRIM reflecting the level of damage that occurred in an SF14 cube with a 100 nm side over 1 day, i.e., 1.6 × 10^−2^ dpa. (**a**) ^237^Np recoil atoms (blue dots) and alpha particle paths (orange dots). (**b**) Recoil cascades (red dots) and ^237^Np recoil atoms (blue dots).

Figure 1 shows a 3D representation of alpha-recoil cascades into a volume of 10^6^ nm^3^ generated in one day for the SF14 sample. The cascades were calculated using SRIM2013 [4] and a macro written to allow for a space and time distribution of the cascades. The path (blue fading to white at the end of range) of the recoil nucleus, ^237^Np, from a decaying ^241^Am is represented in Figure 1a as well as the corresponding alpha particle (orange). In Figure 1b the path of the ^237^Np recoil is again represented in blue, while the red spots are the displaced atoms from the recoil cascades. For the calculation only U, Np, Pu, Am, and O atoms were included and a mean displacement energy of 40 eV for the cations and 20 eV for O were used [5]. These values, as used in the Kinchin–Pease approach [6] to determine the damage as calculated with SRIM, were not precisely assessed for all the actinides, but were generally taken as close to those for U and O in UO_2_. It was, for example, shown that for Pu in (U, Pu)O_2_ the values could be slightly lower [7], as determined by the molecular dynamic calculation of the cascades.

^238^Pu has been used over many years in several studies to accelerate damage accumulation in materials studied for the immobilization of high-level waste or for the burning of excess plutonium in nuclear fuel [8,9,10]. In their paper, Burakov et al. studied immobilization matrices such as monazite, pyrochlore, zircon, or cubic zirconia doped with ^238^Pu (same structure as UO_2_), which showed a good resistance to damage by a competing process of damage build-up and annealing processes, whereas in PuO_2_ (with approximately 10 at% ^238^Pu) it was less evident that annealing occurred [11]. More studies have been performed with ^238^Pu-doped UO_2_ synthesized to mimic nuclear spent fuel for the same purpose of accelerating the damage accumulation. Some of the observations made are reported here to cover other damage ranges and/or different compositions than the SUPERFACT samples. For these ^238^Pu-doped UO_2_ samples, several investigations were reported in [12,13,14,15,16,17,18]. Among the properties studied in the aforementioned works were lattice parameter evolution (an increase of 0.4% until saturation can be anticipated), microstructure evolution (formation of dislocation loops increasing in size and density), the increase in Vickers hardness, the formation of nanometric helium bubbles, and the decrease in thermal conductivity. For these properties, it could be shown that the linearity applies for systems having similar alpha activity to spent fuels, and to some with activities even two orders of magnitude higher. In this paper, we have extended the study to systems (i.e., the SUPERFACT samples) with high alpha activity due to their even larger minor actinide content, among them ^241^Am.

**Figure 2:**
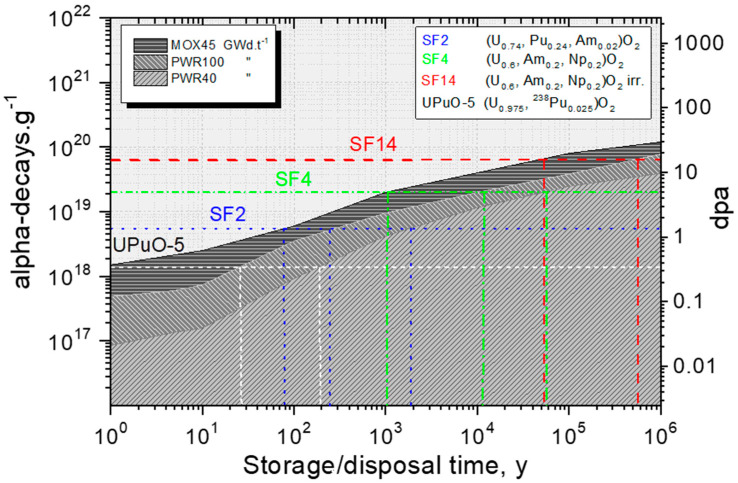
Evolution of the alpha dose and damage (expressed as dpa) in three types of fuels: PWR fuel with 40 GWd.t^−1^, high burnup PWR fuel with 100 GWd.t^−1^_HM_, and MOX with 45 GWd.t^−1^. The figure also shows the equivalent doses/damage of four of the samples of this study that will be discussed in more detail in the Materials and Methods section.

The cumulated damage at the time of investigation and the equivalent storage time for different types of fuels are shown in Figure 2 and reported in Table 1 (for better readability).

## 2. Materials and Methods

### 2.1. Sample Production (SF2, SF4; Archive, ^238^Pu-Doped UO_2_)

All the SUPERFACT fuels were prepared by a combination of gel-supported precipitation (GSP) and powder processing methods [19]. The feed stock was uranyl nitrate and the plutonium, americium, and neptunium dioxides, which were available as powders. Uranyl nitrate was dissolved in water, while nitric acid (ca. 3M) was the solvent for AmO_2_ as well as for PuO_2_ and NpO_2_, at a higher concentration (14M) in the latter case. The stock solutions were analysed and mixed as specified by the chemical composition of the desired fuel. The external gelation route was the GSP process used for SUPERFACT.

The most critical step was to increase the viscosity of the solution by the adding specific thickeners. The resultant broth solution was then passed through a dispersion device to prepare droplets via a high-speed rotating cup, which were collected in an ammonia bath. Once dried, the obtained particles were calcined to provide the dioxide feed powders.

Further processing of the fuel followed conventional compaction and sintering steps. Compaction was performed using a Bussmann uniaxial hydraulic press, furnished with a floating die to minimise pressure and avoid density in-homogeneities through the sample. The furnace was a ceramic tube furnace. As expected, auto-radiographies confirmed an (almost) uniform distribution of the actinides in all fuels. The pellets had a diameter of approximately 5.4 mm and a density of approximately 96% of the theoretical density. Homogeneous grain sizes were observed by ceramography, and autoradiography confirmed a rather homogeneous MA distribution.

The (U_x_, Pu_1−x_)O_2_ samples were prepared according to a similar sol–gel process described in [14,19].

**Figure A1:**
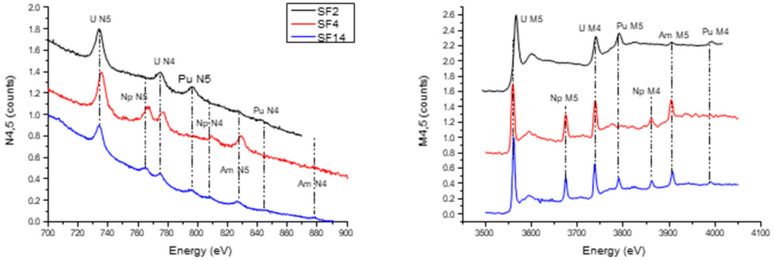
Different regions of the EELS spectrum for each SUPERFACT sample; the M_4,5_ and N_4,5_ edges (used for quantification) for each element are indicated.

All the samples were prepared by a sol–gel process, resulting in a homogeneous distribution of the elements. Some details, such as on the isotopic composition of the various samples, can be found in Table 2, and were particularly important for determining the total cumulated doses in our samples. The elemental composition of the SUPERFACT samples was evaluated by EELS and compared with the initial composition (see Section 2.4 and Figure A1 in Appendix A) or for the irradiated material (SF14) with the calculated theoretical composition:

1. High burnup.
2. Sample irradiated in the Phénix reactor.

### 2.2. Irradiation Condition of SF14

SUPERFACT-1 irradiation took place in the Phénix fast reactor from October 1986 to January 1988 in the same outer position (4th crown) of the internal core of the reactor. The sample used in this study (SF14) originated before irradiation, from the same batch as SF4. The irradiation duration was of 382 Equivalent Full Power Days (EFPD) with a nominal flux of 5.8 × 10^19^ n.m^−2^s^−1^, hence a fluence of 2 × 10^27^ n.m^−2^ at the end of irradiation. The equivalent burnup achieved was 4.1 at% (38 GWd/t_HM_) and the Ti-Ti15 clad reached a dose of 52 dpa. The clad temperature remained in the range of 570–640 °C and the maximal centre temperature was 1920 °C. The linear heat rate ranged from 180 to 290 W.cm^−1^.

**Figure 3:**
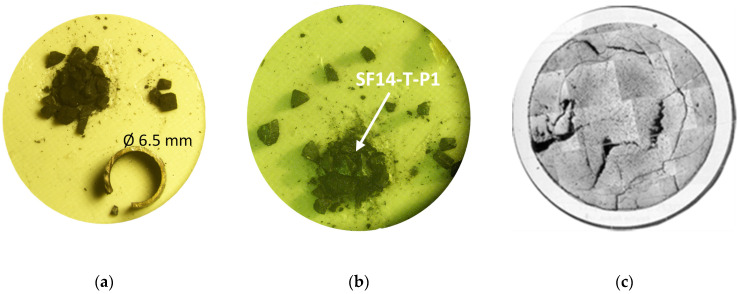
(**a**,**b**) Optical image of de-cladded fuel and the fragment used (SF14-T-P1) in the reported analyses pointed by an arrow, and (**c**) ceramography of a section of the same rodlet.

The actinide transmutation rate was 31% [20]. The beginning of Pellet Clad Mechanical Interaction (PCMI) was noted, and a very high helium production (60 times standard pins) and release (as suggested by high porosity) was measured. The swelling (axial expansion 2.3%, radial expansion 3.3%) was moderated and no restructuring (low power) was observed, which normally occurs in fast reactor fuels, as can be seen on the ceramography in Figure 3c. The irradiated pins were studied by various post-irradiation examinations and the results compiled in a joint CEA–JRC report [21].

The fuel examined by TEM was sampled from a slice cut in an irradiated rod. The cladding was sawed on one side and spread; as the fuel slice that was falling apart maintained its shape, some fragments from an identified radial position could be retrieved. Figure 3a shows a picture taken in the hot cells using a periscope from such a slice of cladding plus fuel, and Figure 3b shows the fragments that constituted the fuel piece in their almost original location. In Figure 3b, the arrow indicates the fragment with the reference SF14-T-P1, which was for the TEM analysis and which corresponded to a position at the centre of the pellet, i.e., irradiated at high temperature (the cladding temperature at end of irradiation was close to 600 °C). In Figure 3c, a ceramography from a cut that was close to the sampled specimen shown in Figure 3a revealed that no typical central void formed during the irradiation in fast reactor conditions (the linear power remained low because of the low fissile content).

### 2.3. Instruments

For the present work, we used a FEI Tecnai G2 TEM, equipped with a GATAN Tridiem camera and a GATAN Imaging Filter. The accelerating voltage for the field emission gun was 200 kV.

To examine highly active or irradiated nuclear materials, the TEM had to be modified by mounting a glove box around the compustage [22]. In this way, the sample could be transferred from the glove box where it was prepared with the microscope by a La Calhène DPTE^®^ system.

For the TEM analyses, the samples were lightly grounded from fragments initially collected on the irradiated material or from archival material. A single-tilt TEM holder was used so that the examination could not result in the possibility of selecting a specific zone axis. This was important for the identification and quantitative determination of dislocation loops with preferential habit planes. Therefore, no conclusions were made on quantitative aspects (dislocation loop concentration).

The elemental and isotopic analyses were performed after a synthesis of the various types of fuels. In the present work, we used EELS M4 and M5 edges to calculate the ratios between the different actinides present in each sample, using the methodology described in [23]. The results are presented in Section 3 and the original spectra in Appendix A.

The Scanning Electron Microscopy work was performed by using a nuclearized Philips^TM^ XL40 and a JEOL™ JM 6300 installed in a shielded hot cell [22].

### 2.4. Decay, Doses, Damage

The samples and their damage dose are listed in Table 1. The NUCLEONICA decay engine [24] was used to calculate the total number of alpha decays from the date of last annealing of the samples until their examination by TEM. The initial isotopic composition was known for the precursors of the different samples and of the irradiated material, SF14, as determined by EPMA [20]. The values of the SUPERFACT samples are reported in Table 2, with the measurement of the elemental composition assessed by EELS.

The dpa parameter was used as a measure of the damage expected in the sample, which on this type of material directly relates to the number of alpha decays by the equation:

dpa = (1700 × α)/n(1)

where n is the number of atoms in the sample and α is the number of alpha decays. By running an SRIM simulation of displacements produced by an alpha decay of 5.5 MeV in UO_2_, a multiplying factor of 1700 can be obtained [25] (accounting for the displacements from both the recoil and the alpha particle).

Figure 1 displays 65 alpha decay events taking place in the SF14 sample over one day. Given the error in the SRIM calculation (between 5 and 10%, see [4]) the difference in energy and mass of the recoil from the various actinides was not considered. As mentioned above, displacement energies were assessed for U and O in UO_2_ as 40 eV and 20 eV, respectively [5], and the same values were used by default for the other actinides in this fluorite structure as no measurements had yet been performed.

For the ion-implantations and/or -irradiations discussed later in this paper (most of the damage was studied in UO_2_), we used the correlation between fluence and dpa made by several authors in order to compare their observations with the ones made for our alpha-doped samples. The doping in minor actinides results in a anisotropic distribution of the damage, whereas implantations/irradiations create a damage zone mostly located around the end of the range of the considered ion, corresponding to mainly nuclear energy losses [26]. The TEM observations carried out on implanted samples took place in the highest damaged areas. For example, in the work of Haddad et al. [27], the damage created in UO_2_ implanted with 260 keV Xe-ions at doses from 10^13^ to 4 × 10^15^ ions cm^−2^ corresponded, on average, to 0.11 to 44 dpa, respectively. In the work of Sabathier et al. [28], the investigated TEM areas of 100 nm thick foils irradiated with 2 × 10^16^ Au-ions cm^−2^ of 4 MeV (initial energy) presented a damage of 1.6 dpa (in this case, the energy loss still had a high electronic contribution resulting in less displacements). He et al. [29] analysed an area implanted with 150 keV (initial energy) of 5 × 10^15^ Kr-ions cm^−2^ by TEM, which presented an average damage of 13 dpa. This last value can be compared with the example of helium implantation. In their work, Belhabib et al. [30] calculated a damage of 0.025 dpa for 10^15^ He-ions cm^−2^ with 50 keV energy. As a last example, one could cite the recent work of Bricout et al. [31] that estimated a damage of 1 dpa in the TEM-investigated area, generated from an initial 900 keV I-ions at a dose of 5 × 10^14^ ions cm^−2^.

As more studies were performed on ion-implanted/ion-irradiated samples than on doped materials, the agreement between the mentioned damages, expressed in dpa, is important in terms of comparing the radiation effects in the two types of samples. It should also be noted that typical dose rates for damage production are approximately two orders of magnitude larger for implantation than in for doped, self-damaged materials.

The third type of sample briefly discussed in this paper is irradiated LWR fuels. Alongside the very large damage accumulated during reactor irradiation (approximately 1 dpa per day), it is recognized that additional alpha damage is generated during storage due to the presence of actinides that are already in the material and produced during irradiation [32].

## 3. Results

### 3.1. Elemental Evolution of the SUPERFACT Samples with Time: EELS Analysis

The first of the studied samples, SF2 had a relatively low content of Am and a high content of Pu (24 at% Pu MOX with 2 at% Am) material. This sample was not irradiated, and thus the storage time was slightly longer than the irradiated one. The calculated alpha damage was 1.3 dpa; although a large amount of Pu was present, the majority of it was ^239^Pu, with a very long half-life (24,100 a), so it did not significantly contribute to alpha damage. ^240^Pu is also not very alpha active as its half-life is 6561 a. ^241^Pu decays to ^241^Am by beta, and thus the alpha damage from this decay was zero. Finally, ^242^Pu is almost stable, with a half-life of 373,500 a. Overall, among all the plutonium isotopes, only the 0.6 at% ^238^Pu would contribute extensively to alpha damage. Thus, the damage was mainly produced by ^241^Am (original and from ^241^Pu decay) and ^238^Pu.

The second sample, SF4, contained approximately 20 at% Am and 20 at% Np. The composition barely changed after 30 a of storage, but almost all the damage came from ^241^Am decaying to ^237^Np because the former isotope has a relatively short half-life and consequently a relatively high activity. As shown in Table 2, the change from the decayed ^241^Am into ^237^Np did not introduce new isotopes in the composition. Due to the high content in ^241^Am, the cumulated damage in this sample was higher than the previous, reaching 4.9 dpa.

The third sample, SF14, was the same as the previously described sample (SF4), but studied after irradiation in the Phénix reactor. During irradiation, the elemental isotopic composition changed, producing a variety of isotopes. Among these newly produced actinides, ^238^Pu would produce most of the damage due to its short half-life (89 y), and ^242^Am (with a short half-life of only 16 h) decays to ^238^Pu, increasing the effect of this isotope. Due to the high temperature reached during irradiation, all the possible alpha damage accumulated between the production date and the finish of the irradiation was annealed and all the helium associated released. Notwithstanding, the alpha activity of the ^242^Am and the ^238^Pu, together with the ^241^Am and ^237^Np (and the ^241^Am created by the decay of ^241^Pu via β), led to an accumulated damage of 15.9 dpa.

### 3.2. SEM Observations

**Figure 4:**
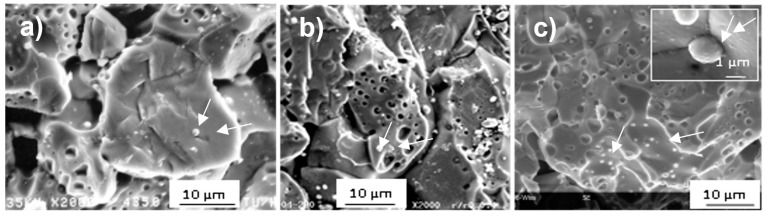
Secondary image of SF14 recorded in (**a**) 1991, (**b**) 2003, and (**c**) 2014. (N.B. the samples SF2 and SF4 were not examined by SEM).

The 2014 SEM examination of the irradiated sample SF14 did not reveal any changes compared to the first examinations that took place during the post-irradiation examinations in 1991, 3 years after the reactor discharge of the samples, and in 2003, 15 years after the discharge. Figure 4a–c shows the SEM images of an area of the irradiated target, which reveals the large porosity that formed, partly attributed to the large quantity of helium formed during irradiation.

The small spherical precipitates that can be observed in the three images in Figure 4 (indicated by arrows), highlighted in the inset of Figure 4c, are the typical noble metal (Mo, Tc, Ru, Pd, Rh) fission product precipitates formed during irradiation. Their size, on average close to 1 µm, derives from the high irradiation temperature [33], as was also observed in LWR fuels irradiated at high temperature (see, for example, [34]).

Some of the ^238^Pu-doped samples were periodically observed by SEM and no alteration of the microstructure was observed [35].

### 3.3. TEM Observations

The work presented in this paper focuses on the TEM observations performed on the different samples to assess the microstructure as a function of cumulated alpha doses for which equivalent storage times of real spent fuels are listed in Table 1. The main observations that were obtained from the microstructure examinations are detailed in the next subsections.

#### 3.3.1. SF2–1.3 Dpa

**Figure 5:**
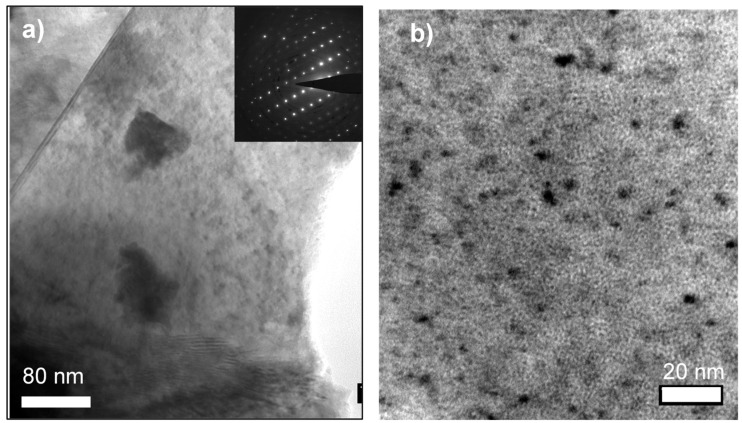
TEM bright field micrographs of the SF2 sample (1.3 dpa) showing: (**a**) Flat area oriented by the [110] axis showing dislocation loops (dark, circular marks); inset image is the electron diffraction proving the orientation. (**b**) Higher magnification of dislocation loops with sizes ranging from several nanometers up to ten nanometers (dark marks), together with nanometric over-focused (dark, round spots) and under-focused (light, round spots) helium bubbles.

The alpha damage on the first sample of study, i.e., SF2 (1.3 dpa), consisted in a large number of dislocation loops. These dislocation loops were found on every particle studied, with sizes that ranged from 5 to 10 nm (some even larger). Figure 5a,b shows TEM micrographs where dislocation loops can be observed as dark disks. In Figure 5b, helium nano-bubbles can be observed as bright spots typically visible in under-focused conditions. It should be noted that the size of the bubbles looks much larger under these conditions than they were, and their concentration correspondingly smaller. An analogy can be made with the recent study of Onofri et al. [36] showing that, in the case of nano voids, their size and density can vary substantially as a function of the TEM settings.

#### 3.3.2. SF4–4.9 Dpa

**Figure 6:**
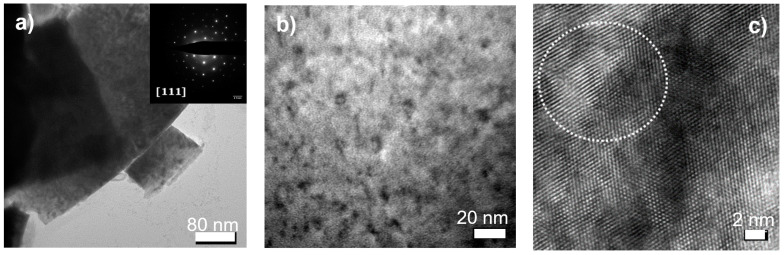
TEM bright field image (**a**,**b**) of sample SF4 (4.9 dpa) showing dislocation loops habiting the [111] plane, as shown by the electron diffraction in the inset of (**a**). (**c**) HRTEM image showing a single loop of approximately 5 nm diameter (circled by white dots).

In this archive sample, with initially 20 at% Am and 20 at% Np, the accumulated damage consisted of dislocation loops and some small dislocation line segments, as can be seen in Figure 6a,b. The size of the loops is larger than in the SF2 samples, while their concentration appears slightly lower. Figure 6c shows, for example, an image in high resolution of a loop with a diameter of 5 nm. The total damage of almost 5 dpa was substantial, and nanometric bubbles are also observed (white dots on Figure 6b).

#### 3.3.3. SF14 Irradiated–15.9 Dpa

**Figure 7:**
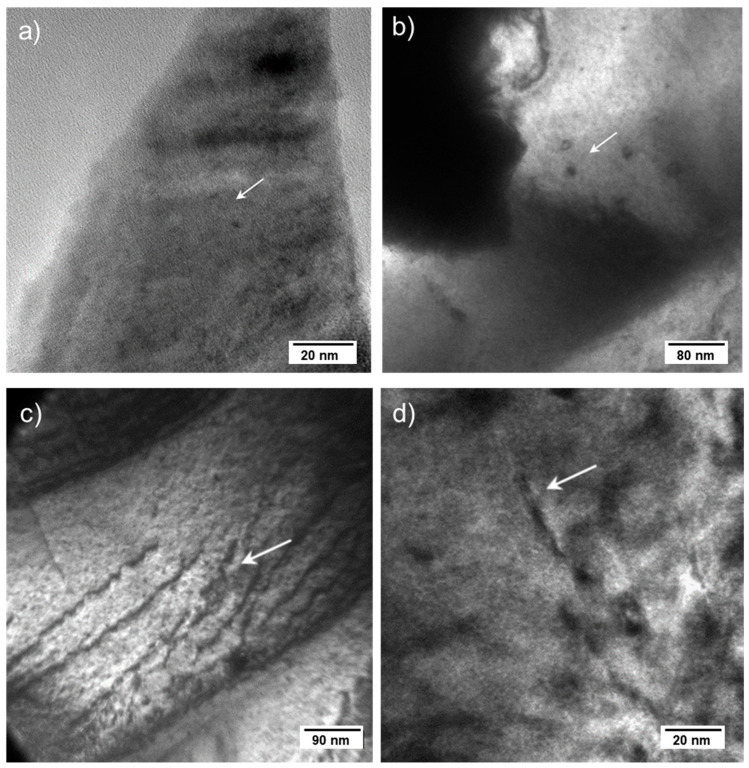
TEM bright field micrographs of sample SF14 (15.9 dpa) showing (**a**,**b**) dislocation loops, (**c**) dislocation lines, (**d**) dislocations and helium nanometric bubbles (bright spots). All mentioned features are indicated by arrows in the corresponding images.

In the irradiated sample that cumulated almost 16 dpa (after irradiation), some larger dislocation loops were visible (Figure 7a,b), while dislocation lines were also present, as can be seen in Figure 7c. In addition, nanometric bubbles were also present, as can be seen in Figure 7d. The total number of displacements accumulated during irradiation in the reactor could amount to a value as high as 1500 dpa (1 dpa/day) by considering only the displacements produced by elastic collisions from the fission fragments. The dislocation lines can be formed during irradiation and, in particular, at high temperature where visco-plastic deformation can occur, the observation of dislocations is expected.

#### 3.3.4. UPuO-5–0.34 Dpa

**Figure 8:**
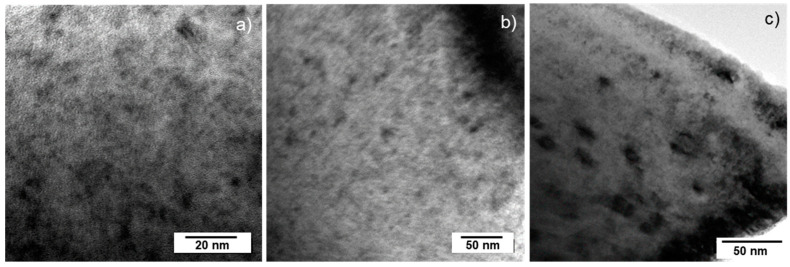
TEM bright field micrographs of sample UPuO-5 (0.34 dpa) showing (**a**,**b**) 3–7 nm dislocation loops (**c**) 20 nm dislocation loops.

In this sample, which was examined 450 days after production and had cumulated approximately 0.34 dpa, microstructure analysis revealed the presence of dislocation loops with sizes between 3 and 7 nm, and in some cases as large as 20 nm, as shown in Figure 8a–c and as also recently partly reported in [18]. Some further properties (thermal diffusivity, lattice parameter) were measured and reported for this sample recently [14,18]. It could be shown that the microstructure was evolving from an early formation of dislocation loops to larger dislocation loops, reducing in density with increasing dose. The equivalent storage time of a standard LWR fuel would be around 200 years for this sample at the time of this study (see Table 1).

#### 3.3.5. UPuO-10–4 Dpa

**Figure 9:**
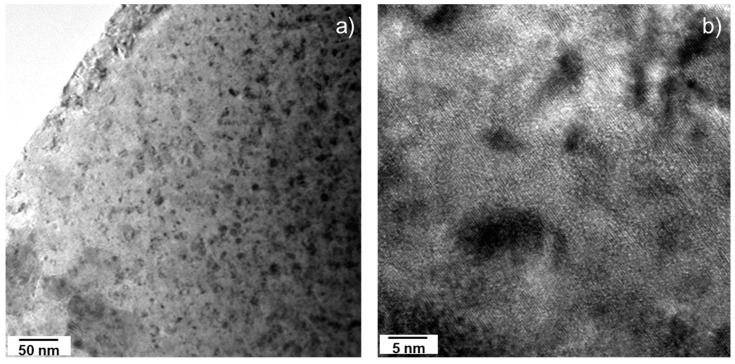
TEM bright field micrographs of sample UPuO-10 (4 dpa) showing (**a**) dislocation loops of 5 nm or more, and (**b**) details on higher magnification showing nanometric and sub-nanometric helium bubbles (white spots) and defect (interstitial) clusters (small black dots of 1–2 nm).

The TEM micrographs shown in Figure 9a,b from the ^238^Pu-doped UO_2_ sample were collected at a damage level of 4 dpa. This level of damage is expected after about 30,000 years of storage for a standard LWR fuel. Several results from the microstructure analyses have been reported for this compound [3,13], showing its potential use as a spent fuel surrogate. In the paper of Jonnet et al. [17], a mechanism of coalescence of diffusing interstitial-type dislocation loops was proposed to justify an increase in the size of the loops, which for 4 dpa reach a typical value of 5 nm compared to 3 and 3.6 nm for 1.1 and 2 dpa, respectively. The dopant concentration (^238^Pu) resulted in an activity of 3.76 × 10^10^ Bq.g^−1^, from which the damage rate was found to be 9 × 10^−9^ dpa.s^−1^. Sub-nanometric helium bubbles were observed for the sample with 4 dpa.

#### 3.3.6. Irradiated UO_2_ Fuels–0.1 Dpa

In this section, we intend to show that an irradiated fuel microstructure, whatever the experimental conditions (light water reactor vs. fast reactor technology), can share some similarities with alpha-doped materials.

**Figure 10:**
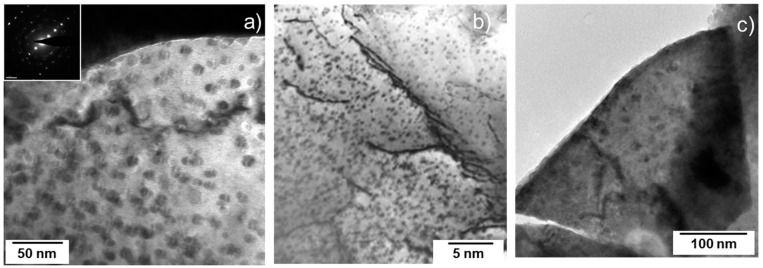
TEM bright field micrographs of UO_2_ fuel irradiated at; (**a**) 70 GWd.t^−1^; (**b**) 34 GWd.t^−1^, and (**c**) 58 GWd.t^−1^. For all samples, the cooling before examination led to the accumulation of approximately 0.1 dpa.

In a very similar approach to the SUPERFACT SF14 sample case, the dpa considered here were accounted only after irradiation. In the case of the examined LWR fuels, it was in the order of 0.1 dpa, i.e., much lower than SUPERFACT SF14 (15.9 dpa). However, the nature of the damage observed was very similar to what is observed in alpha-doped materials. The size of the observed loops was very close to 10 nm, which is larger than what is observed in the alpha-doped materials examined here. In addition to dislocation loops, one can also observe some dislocation lines and fission gas bubbles. Evidently, the microstructure is not solely due to the alpha damage accumulated during storage but also from the irradiation. Indeed, dislocation lines resulting from irradiation are observed in most irradiated fuels and their presence is attributed to precipitation and the interaction of loops, or to thermo-mechanical stresses. However, no TEM examinations took place at the discharge of any irradiated fuel and the annealing of the dislocation loop was found in alpha-doped UO_2_ at a temperature of 900 K. The three fuels, for which TEM micrographs are shown in Figure 10a–c, were irradiated at temperatures higher than 900 K and revealed the presence of dislocation loops. It should be noted that the linear power, reactor shut down and scram, and the radial position (hence, temperature experienced) all play a role in the microstructure of the fuel at discharge, but it is outside the scope of this paper to discuss these aspects. Some general description of the fuel microstructure as a function of burnup and irradiation temperatures can be found in [34,37]. It can be argued that the nature of TEM-observable (extended) defects is very similar in stored irradiated fuel and alpha-damaged actinide dioxides, except for dislocation lines.

## 4. Discussion

This study is focused on microstructure evolution as a function of the cumulated alpha damage formed in spent fuel surrogates, and it complements the assessments made on kinetic effects from alpha damage and, in particular, from studies of ^238^Pu-doped UO_2_ [18,38]. Both of those works showed that within the range of initial alpha activity studied there is a linearity in the measured properties (hardness, lattice parameter increase, thermal diffusivity, etc.), meaning that there is no dose rate effect, at least for the specific activities under study.

The main microstructure characteristics of alpha-damaged actinide dioxides with a fluorite structure consist of the formation of dislocation loops as extended defects whose size distribution and density vary with the accumulated alpha dose [13,17,25,36,39].

The dislocation loops’ size and density for the samples of this study were not assessed, due to the way the samples were prepared, i.e., by the crushing of small fragment. These samples were not suitable for a double-tilt TEM sample holder due to the risk of losing a radioparticle in the column, hence crystallographic analyses of the loops’ characteristics (Burgers vector, habit plane) become extremely difficult. Indeed, a certain fraction of the loops could be unaccounted for. It is nevertheless considered that the most probable habit plane would be {110}, and to a lesser extent {111} for UO_2_. This has been studied, for example, in the work of Onofri et al. [40] and by Bawane et al. [41] for proton irradiated ThO_2_ showing a more frequent loop precipitation on the {111} plane.

Indeed, another approach used to investigate the formation of damage in actinide dioxides, and UO_2_ in particular, is to make use of accelerated ion beams. For the case of alpha damage, it occurs via implantations/irradiations with helium beams (the alpha particle) or with heavy atoms at energies of approximately a few hundred kiloelectronvolts to simulate the recoil nucleus. Some of these works will be described to make comparisons with the results obtained in this study. It should be emphasized that the dose rate from accelerated beams is an order of magnitude one hundred times larger than those from alpha-doped materials. At certain alpha doses, whether in ion (He)-irradiated materials or in alpha-doped materials, helium bubbles can be observed by TEM. As a very rough conversion factor, one can assume an equivalence of 5 × 10^18^ He-at. g^−1^ in alpha-doped materials for 0.1 at% in implanted specimens. Several implantation studies of helium in UO_2_ were performed and bubbles were observed [42,43,44,45]. Recently, A. Michel performed a thorough TEM investigation of the He bubble precipitation in UO_2_ [46]. The author implanted two specimens at 7 × 10^16^ He-ions cm^−2^ at 200 °C and at a fluence of 3 × 10^17^ at.cm^−2^ at 700 °C, resulting in a concentration of 0.03 and 0.15 at% corresponding to 0.05 and 0.24 dpa, respectively, at the implantation depth, therefore close to our sample, UPuO-5. The main conclusion of his work was related to helium bubbles and focused ion beam artefacts being the possible interferences for the measurement with features below 0.4 nm. However, it was demonstrated that no (visible) helium bubbles formed at a concentration of 0.05 at% in (U_0.8_, Pu_0.2_)O_2_ single crystals [46]. The particular aspect of helium bubbles is not discussed here, particularly when studied on ion-implanted samples, i.e., only the alpha particle effect. It is more probable that a heterogeneous precipitation of bubbles is predominant in alpha-doped (and reactor-irradiated) samples due to the formation of defects by the recoil nucleus in particular. In addition, it can be noted that the alpha particle does not generate a relevant amount of displacements compared to the recoil nucleus, as previously mentioned in Section 2.4.

The damage aspect, which was the main point of focus of this study, can be supported by some observations made on ion-implanted/-irradiated samples, typically for UO_2_. The main defects observed by TEM in our study consisted of dislocation loops as described in Section 3. The dislocation evolution of a UO_2_ single crystal implanted with Kr was studied by He et al. [29] who showed that, whereas low irradiation doses result in nucleation and the growth of dislocation loops, high doses cause a transformation into large dislocation lines and networks, as well as a shrinkage and even a disappearance of some dislocations and loops due to high temperature annealing. In general, the behaviour of dislocations in Kr irradiated UO_2_ was similar for both combinations of ion energy and temperature they used (150 keV at 600 °C and 1 MeV at 800 °C).

The highest dislocation loop density measured was around 5 × 10^14^ ions cm^−2^. Some dislocation loops started to transform into dislocation segments around 1 × 10^15^ ions cm^−2^ (13 dpa), eventually evolving into tangled networks at higher doses through coalescence/coarsening mechanisms, as was also observed at 44 dpa by Haddad et al. [27]. This observation is in agreement with the microstructure observed in SF14, i.e., at 16 dpa, where some dislocation loops co-exist with dislocation segments, probably resulting from coalescence.

Onofri et al. [40] irradiated polycrystalline thin foils with either 4 MeV Au- or 390 keV Xe-ions at different temperatures (25, 600, and 800 °C) and fluences (0.5 and 1 × 10^15^ ions cm^−2^) to fully characterize both dislocation loops and lines induced by ion-irradiations in UO_2_. TEM analysis was employed to determine their Burgers vectors, habit/slip planes, and interstitial or vacancy nature.

Their investigation, carried out on a large number of dislocation loops sized between 10 to 80 nm, as well as for the first time on a number of dislocation lines, discovered unfaulty prismatic dislocation loops of interstitial nature, with Burgers vectors positioned along the <110> directions only.

In a later work, in situ TEM analysis was employed by Onofri et al. [47] to study the effect of temperature on dislocations in pre-irradiated, polycrystalline UO_2_ foils.

Whereas initially dislocation loops were only observed due to low temperature and low fluence irradiation, a second set of small (<5 nm in diameter) dislocation loops appeared at around 500 °C. The cause may be the dislocations acting as sinks for point–defect recovery. In the case of both dislocation lines and irradiation-induced loops being initially present, strong line rearrangements by slipping took place, particularly around 800 °C. Then, regardless of the initial irradiation conditions, a second annealing phase related to extended defect recovery was detected at above 1000–1100 °C.

The density of dislocations, both of the loop and line variety, decreased with increasing temperature.

In the recent work of Onofri et al. [47], the temperature effect was clearly demonstrated. A direct correlation with our observations was made on a purely qualitative aspect. The damage level in the thin foil irradiated by Onofri et al. was in the order of magnitude of that observed in the irradiated LWR fuels, i.e., 0.1 dpa. The evolution of dislocation loops into dislocation lines at 500 °C supports our assumption that the extended defects produced at irradiation at 630 °C would consist mainly of dislocation lines and that the observed dislocation loops are formed during storage.

In the case of alpha decay, there is a mix of elastic and inelastic energy loss from the recoil nucleus and alpha particle, respectively, compared to ion-irradiations. However, recently, ion-irradiation experiments attempted to discriminate between the contributions of both (sequentially and simultaneously) to the formation of defects.

Following the irradiation by two different beams (900 keV I and 27 MeV Fe, hereafter referred to as Se and Sn, respectively), both a lower point defect density and lower strain levels were observed; nevertheless, TEM results pointed out that electronic ionizations modify the defects generated by nuclear collisions [31]. An evolution from dislocation loops generated by single Sn irradiation to dislocation lines in the case of dual beam irradiation is indeed found for a similar Sn fluence.

The lattice appears to heat up, through an electron–phonon coupling, enough to increase local defect mobility. The next step would be for dislocation loops to trap smaller defects and therefore increase their size until dislocation lines originate. These findings point to a modification of the UO_2_ matrix, induced by the combination of electronic energy loss and defects created by nuclear energy loss. [31]. This study reported, for the first time, the concomitant effect of Sn and Se using implantation. However, the ratio between Sn and Se in alpha decay remains rather low, so that the Sn effect should remain dominant. Indeed, in the study by Bricout et al. [31], the Sn/Se ratio was approximately 0.23, whereas for alpha decay, this ratio was approximately Sn/Se = 0.05. It is not expected that alpha particles can cause any substantial displacement cascades, but rather by thermal spike effect in UO_2_ they promote the diffusion of defects in addition to the displacement cascades generated by the recoil nucleus. The study by Ferry et al. [48] on the self-alpha irradiation-induced diffusion of oxygen and uranium describes the thermal effects and displacement cascades from the recoil nucleus as both having a contribution to the mobility of uranium and oxygen, and so we can assume that it could promote some growth of dislocation loops.

Primary radiation damage in(U_1−y_,Pu_y_)O_2_ solid solution was also studied through molecular dynamics simulations for various temperatures and plutonium contents [7,49].

This assessment was performed in four parts: (a) defect formation energies, (b) Frenkel pair recombination, (c) displacement cascades, and (d) modelling the dose effect by the Frenkel pair accumulation method.

Primary damage behaves in the same way with respect to increasing the dose, as previously observed in pure UO_2_. First, point defects are created. They eventually cluster and form small Frank loops, which in turn grow into unfaulty loops. The study also put into evidence the effects of plutonium content, showing that dislocation density decreases as plutonium content increases when using the Cooper potential, due to a higher degree of Frenkel pair recombination [7]. During irradiation in the reactor, the fuels cumulate a very high level of damage from the fission spikes [25]. Point defects, as well as extended defects (dislocation loops and lines) are formed and evolve as a function of the irradiation temperature in different microstructure, including the so-called High Burnup Structure (HBS), evidently associated to the formation of large amounts of defects [37,50,51,52].

In order to analyse nuclear fuel reaching the end of its in-pile life, no examination can take place in a hot cell before the fuel has cooled down. Since the irradiated fuel has a residual large alpha activity component, to assess its properties when examined out of pile, the material degradation that occurs during cooling has to be taken into account. Alpha-doped samples are very valuable in this respect, since, in their case, the effect of low-temperature cumulated alpha-decay damage can be independently studied [22].

G. Brindelle clearly evidenced that, during storage, an increase in alpha damage (three thermal releases were performed on samples from the same fuel but stored for different times) impacts the release behaviour of xenon during thermal annealing [32]. This finding confirms that the fuel is not in a steady state and that the damage evolves over a time scale of only a few years. The exact nature of the defects, although not studied by TEM, nevertheless confirm that defects responsible for migration (in these cases, gases) are produced and would therefore also certainly contribute to the evolution of the spent fuel [32].

Haddad et al. performed implantations of UO_2_ single crystals with 470 keV Xe or 500 keV La at 773 K and fluences between 10^13^ and 10^16^ ions cm^−2^ to investigate, through Rutherford Backscattering in Channelling mode (RBS-C), how radiation damage and the incorporation of extraneous elements affect matrix behaviour. In situ TEM images show the appearance and evolution of several defects as a function of the ion dose up to 44 dpa, regardless of the chemical nature of the bombarding ion: ‘black dot’ defects, dislocation loops and lines at first, then a dislocation network at a higher dpa [27].

These results from the ion-implantation studies can be compared to our results. The basic mechanisms of damage formation, whether produced by implantation or by decay, will be of the same nature providing the energy and mass of the considered ion are the same, or in better words the energy loss is the same. For the recoil nucleus, it is predominantly nuclear, whereas for the alpha particle, the electronic energy losses are predominant [26]. The type of residual defects and their evolution are dependent on dose rate, temperature, elemental composition, and stoichiometry of the compound. The current study showed that (observable) defects produced by alpha decays in actinide oxides of different composition are of the same nature (dislocation loops and lines) within the studied dose rates. A thorough description of the evolution of the defects would require integrating the point defects. This can be obtained from X-ray diffraction data (lattice parameter), which are impacted by the two classes of defects (point and extended). The results of a former study on the defects formed at low damage level, and their impact on thermal diffusivity has been previously published [53]. In the specific case of the SUPERFACT samples studied in this paper, the damage that has been reached (more than 1 dpa) is beyond the threshold for which the saturation (increases between approximately 0.3 and 0.4%) of the lattice parameter is observed for several actinide oxide compounds [12,13,18,54,55,56,57,58]. The steady state in the creation and annealing of point defects has been the main explanation of the saturation of the lattice parameter. The kinetic aspects are of importance, and it has been shown recently that within the dose ranges studied here the behaviour was linear [18]. After saturation of the lattice parameter (at approximately 1 dpa), an increase in dislocation loops size was observed, and it could be demonstrated that while strain increased, the micro-strain decreased in alpha-doped samples (isotropic damage).

In particular, in our study the SF4 sample and UPuO-10, with 4.9 and 4 dpa, respectively, compared very well to each other. This qualitative assessment of the self-damage associated to alpha decay in materials definitely needs more precise quantification. Single effect studies such as ion-implantations show a very similar trend in the microstructure evolution of alpha damage in UO_2_ and, in particular, due to the recoil nucleus, they show the main contributor to defect formation, which compares favourably in terms of dpa.

To this extent, the irradiated SUPERFACT SF14 constitutes an extremely highly damaged fuel, equivalent to LWR standard fuels aged by more than 1 Ma, that remain integral despite the amount of radiogenic helium and damage generated. No single effect studies have (yet) combined both helium and equivalent recoil nucleus doses to this extent, therefore, this study constitutes a first approach to predicting the real spent fuel behaviour over long storage times.

The dose rates for the alpha-doped materials, including the SUPERFACT samples, remain low compared to ion-implantations/irradiations. However, they are themselves extremely low compared to the activities of standard LWR fuels, either UO_2_ or MOX fuels. In a previous study [13], we revealed that the microstructure of alpha-doped materials and very old uraninites or thorianites compare well. One of the conclusions of that paper was related to the possible accommodation of radiogenic helium in the fuel microstructure. It was shown from the TEM investigation of the SF14 sample that no evident micro-cracking developed despite very large amounts of helium and defects generated. The SF14 sample was very porous due to the irradiation temperature, and could therefore retain a high quantity of the newly formed helium. No mechanical properties were measured nor XRD performed on the SUPERFACT samples in general, but as evidenced by the recent studies on alpha-doped materials [18], with increasing alpha dose, the micro-strain reduces, most probably because of the saturation of point defect formation and precipitation, growth of dislocation loops and the further saturation of dislocation lines.

## 5. Conclusions

Actinide dioxides with the fluorite structure can be used for the prediction of LWR spent nuclear fuel behaviour over an extended storage time or even over disposal times. The SUPERFACT experiment, performed in the mid-1980s, constituted the first transmutation test of minor actinides in a fast reactor. The materials tested were UO_2_-based ceramics with various amount of the minor actinides to be transmuted (Am, Np).

The observed microstructure presents very strong similarities, in particular regarding the formation of dislocation loops and of nanometric helium bubbles, to what was found by previous studies on ^238^Pu-doped UO_2_ samples, in which the ageing of such fluorite compounds was also accelerated. It was previously demonstrated that for ^238^Pu-doped UO_2_, a difference of a factor of 100 in activity does not affect the linearity of some properties related to damaging effects. The current work shows that microstructure evolution follows a similar profile independently of the studied dose, and thus corroborates this finding and extends the range of materials to study from the ageing of spent LWR fuel to minor actinide doped UO_2_.

The irradiated SUPERFACT transmutation fuel constitutes an even more representative material because it has been irradiated and hence contains fission products and reactor irradiation damage. Despite the similarity with irradiated fuels, it also differs substantially because of the initial (and final) composition in terms of minor actinide content (much higher). The burnup was equivalent to commercial LWR fuel, but the irradiation temperature was much higher, which in turn can constitute a difference in terms of the healing of defects during irradiation, the precipitation of secondary phases, and the behaviour of volatile species.

From the microstructure observations of the different materials of this study, and those made in previous ones, it can be argued that the linear behaviour regarding damage effects could be used to extrapolate the evolution of commercial spent fuel over very long time spans. For the highest dpa studied here, and in particular that of a reactor-irradiated material corresponding to a standard LWR fuel aged more than one million years, it has been shown that the material preserves its integrity and that the microstructure is characterised mainly by the presence of extended defects. Some limitations are intrinsic to the studied materials, whose actinide content does not reflect the reality of real spent fuel, particularly in the case of the irradiated SUPERFACT SF14 fuel, which presented a relatively large porosity formed during irradiation that could accommodate a substantial amount of helium.

## Figures and Tables

**Table 1:** Samples studied with the cumulated alpha dose and damage and their equivalent storage time for several types of LWR fuels.

Sample	α-Decays [g^−1^]	Dpa	Eq. Standard LWR (40 GWd/t)	Eq. HBU LWR (100 GWd/t)	Eq. MOX LWR (45 GWd/t)
			Storage Time (Years)		
SF2 (U_0.74_, Pu_0.24_, Am_0.02_)O_2_	5.5 × 10^18^	1.3	2000	250	80
SF4 (U_0.6_, Am_0.2_, Np_0.2_)O_2_	2 × 10^19^	4.9	55,000	12,000	1000
SF14 (U_0.6_, Am_0.2_, Np_0.2_)O_2__irr_	6.4 × 10^19^	15.9	>1,000,000	550,000	60,000
UPuO-10 (U_0.9_, ^238^Pu_0.1_)O_2_	1.6 × 10^19^	4	30,000	5000	600
UPuO-5(U_0.975_, ^238^Pu_0.025_)O_2_	1.4 × 10^18^	0.33	200	25	1

**Table 2:** Elemental and isotopic composition of the three SUPERFACT samples at the starting point of their storage and their theoretical elemental composition at the time of their study, together with the elemental analyses performed with EELS. At the bottom of the table, the dpa are calculated for each of the SUPERFACT samples.

	**(U_0.74_, Pu_0.24_, Am_0.02_)O_2_, SF2, archive**	**Start (31/08/1988)**		**Study (21/10/2015)**		**(U_0.6_, Am_0.2_, Np_0.2_)O_2_, SF4, archive**	**Start (26/10/1986)**		**Study (26/06/2015)**		**(U_0.6_, Am_0.2_, Np_0.2_)O_2_, SF14, irradiated**	**Start (26/10/1986)**		**EOL**	**Study** **(06/05/2015)**	
**Isotope**		at% Isotp.	at% Elmt.	at% Elmt.	EELS		at% Isotp.	at% Elmt.	at% Elmt.	EELS		at% Isotp.	at% Elmt.	at% Isotp.	at% Elmt.	EELS
**^235^** **U**		0.5	74.4	74.5	75		0.2	59.7	59.7	58		0.1	58.5	0.4	57.3	59.5
**^238^** **U**		73.8					59.5					58.3		54.4		
**^238^** **Pu**		0.3	23.5	21.8	22		-	-	-	-		8.1	11.9	8.8	14.3	11
**^239^Pu**		14.4					-					2.8		3.2		
**^240^Pu**		5.5					-					0.2		0.2		
**^241^Pu**		2.1					-					-		0.014		
**^242^Pu**		1.1					-					0.8		0.9		
**^241^Am**		2.2	2.2	3.6	3		19.0	19.0	18.1	17		14.5	15.1	11.3	13.6	14.1
**^242^Am**		-					-					0.6				
**^237^Np**		-	-	0.1	-		21.3	21.3	22.2	25		14.5	14.5	17.1	14.8	15.4
**dpa ^1^**		1.3 dpa					4.9 dpa					15.9 dpa				

^1^ The dpa values were calculated by the theoretical isotopic composition of the sample and may be subject to errors due to their local distribution, in particular for the scale of the TEM samples. For a better reading, particularly for time correspondence with spent fuel, the calculated values were kept without introducing errors. In particular, the sample with the largest possible error (SF14) corresponded to a standard burnup of spent fuel of more than 1 million years. It was rather more important to highlight the time scale than precise values.

## Data Availability

The data presented in this study are available on request from the corresponding author.

## Appendix A. Elemental Evolution of the SUPERFACT Samples with Time: EELS Analysis

The data available for the elemental composition of the SF14 relied mainly on calculations. Advantage was taken of the EELS to assess the composition of the studied samples. The first sample studied, SF2, had a low content of Am and high content of Pu (24 at% Pu MOX with 2 at% Am). This sample was not irradiated, and thus the storage time was slightly longer than the irradiated one. The calculated alpha damage was 1.3 dpa; although a large amount of Pu was present, the majority of it was ^239^Pu with a very long half-life (24,110 y) so it did not contribute largely to the alpha damage. ^240^Pu is also not very alpha active, as the half-life is 6564 y. ^241^Pu decays to ^241^Am by beta, and thus the alpha damage from this decay was zero. Finally, ^242^Pu is almost stable, with a half-life of 375 ka. All in all, among all the plutonium isotopes, only the 0.6 at% ^238^Pu would contribute extensively to alpha damage. The damage is essentially produced by ^241^Am from the original material and decay product from ^241^Pu.

The second sample, SF4, contained 20 at% Am and 20 at% Np (designated Am20Np20). Almost all the damage came from the ^241^Am which decays to ^237^Np, thus the amount of Am was reduced and the Np increased. Furthermore, a small amount of ^235^U decays. Due to the high content in ^241^Am, the cumulated damage in this sample was higher than on the previous, reaching 4.9 dpa.

The third sample, SF14, was the same as the previous sample, SF4, but taken after irradiation in the Phénix reactor. During irradiation, the elemental isotopic composition changed, producing a variety of isotopes. Among them, ^238^Pu would produce the majority of the damage due to its reduced half-life (89 y) and ^242^Am (with a short half-life of only 16 h), which decays to ^238^Pu, increasing the effect of this isotope. Due to the high temperature reached during the irradiation, all alpha damage accumulated between the production date and the end of the irradiation was annealed, and all the helium associated released [2]. Notwithstanding, the alpha activity of the ^242^Am and the ^238^Pu, together with the ^241^Am and ^237^Np (and the ^241^Am created by the decay of ^241^Pu via β) led to an accumulated damage of 15.9 dpa.

The EELS spectra on this sample were very clear, showing the N4,5 edges and M4,5 perfectly separated and allowing us to carry out a semi-quantitative elemental analysis. Table 2 shows the ratios of all the actinides observed, providing a very accurate result.

The O4,5 edges of all the actinides were overlapping, and thus it is was not possible to observe them independently. There was, though, a drift on the position of the edges, which moves from lower values for the first sample to higher on the last one.

The oxygen K line was obtained in order to see if the average oxidation state of the sample could be, to some extent, evaluated. The shape of these spectra was basically identical, except for a small observed drift, which could not be related to any change in properties.

The quantitative analyses are reported in Table 2.
